# Energy Absorption and Limit Velocity of Epoxy Composites Incorporated with Fique Fabric as Ballistic Armor—A Brief Report

**DOI:** 10.3390/polym13162727

**Published:** 2021-08-15

**Authors:** Michelle Souza Oliveira, Fernanda Santos da Luz, Henry Alonso Colorado Lopera, Lucio Fabio Cassiano Nascimento, Fabio da Costa Garcia Filho, Sergio Neves Monteiro

**Affiliations:** 1 Department of Materials Science, Military Institute of Engineering—IME, Praça General Tibúrcio 80, Urca, Rio de Janeiro 22290-270, Brazil; oliveirasmichelle@ime.eb.br (M.S.O.); lucio@ime.eb.br (L.F.C.N.); fabiogarciafilho@gmail.com (F.d.C.G.F.); snevesmonteiro@gmail.com (S.N.M.); 2 CCComposites Laboratory, Universidad de Antioquia—UdeA, Calle 70 n° 52-21, Medellin 050010, Colombia; henry.colorado@udea.udea.edu.co

**Keywords:** fique fabric, epoxy composite, ballistic test, limit velocity, energy absorption, ballistic armor

## Abstract

Polymer composites reinforced with natural fabric have recently been investigated as possible ballistic armor for personal protection against different levels of ammunition. In particular, fabric made of fique fibers, which is extracted from the leaves of the *Furcraea andina*, was applied as reinforcement for polymer composites used in a multilayered armor system (MAS). The superior performance of the fique fabric composites as a second MAS layer motivated this brief report on the determination of the absorbed energy and capability to limit velocity in the stand-alone ballistic tests. The single plates of epoxy composites, which were reinforced with up to 50 vol% of fique fabric, were ballistic tested as targets against 7.62 mm high-speed, ~840 m/s, impact ammunition for the first time. The results were statistically analyzed by the Weibull method and ANOVA. The absorbed energies of the 200–219 J and limit velocities of 202–211 m/s were found statistically similar to the epoxy composites reinforced with the fique fabric from 15 to 50 vol%. Predominantly, these findings are better than those reported for the plain epoxy and aramid fabric (Kevlar^TM^) used as stand-alone plates with the same thickness. Macrocracks in the 15 and 30 vol% fique fabric composites compromise their application as armor plates. The delamination rupture mechanism was revealed by scanning electron microscopy. By contrast, the integrity was maintained in the 40 and 50 vol% composites, ensuring superior ballistic protection compared to the use of Kevlar^TM^.

## 1. Introduction

Synthetic laminates made of ultra-high-molecular-weight polyethylene (UHMWPE), under the trademarks of Dyneema^TM^ and Spectra^TM^, as well as aramid fiber, supplied as Kevlar^TM^ and Twaron^TM^, have been the most common materials used worldwide for bulletproof vests [1,2,3]. Past decades witnessed a surge in research on the possible substitution of polymer composites reinforced with natural fiber/fabric for synthetic laminates [4,5,6,7]. In addition to sustainable issues, natural fiber/fabric composites possess a comparable capacity to dissipate the ballistic energy with cost-effective advantages [8,9,10,11,12,13,14,15]. The ballistic tests in all of these previous studies, as well as in the present investigation, were conducted according to the NIJ standards [16].

Among the numerous natural fabrics that have been investigated as reinforcement for polymer composites in personal ballistic armor, the fique fabric displayed superior performance as a second layer in a multilayered armor system’s (MAS’s) front ceramic plate [17,18]. In addition, previous studies have evaluated the mechanical properties of the fique fabric epoxy composites by means of impact [19] and tensile tests [20]. The results of these studies have shown that the fique fabric acts as reinforcement, since the incorporation of fique fabric up to 60 vol% increases the value of the tensile strength of the composites by more than twice [20]. Likewise, the epoxy composites with up to 40 vol% of fique fabric exhibited an increase of 2.3 times in the amount of impact energy absorbed in the Izod tests [19].

However, the ballistic performance of the fique fabric composite associated with the standard backface signature (BFS) [16] was, on average, not only better than other natural fabrics, but was also better than using Kevlar^TM^ as an MAS second layer with the same thickness [17,18]. Pereira et al. [17] found that polyester composites reinforced with 10, 20 and 30 vol% fique fabric displayed a BFS of 16–20 mm, which is much less than the limit value of 44 mm imposed by the standard as a lethal trauma [16]. Moreover, these BFS values are better than the average 23 mm which has been reported for the MAS with Kevlar^TM^ as the second layer with the same thickness [11]. The mechanisms of rupture of the polyester matrix, as well as the fabric/matrix delamination, and the individual rupture of fique fibers, were revealed by scanning electron microscopy (SEM) [17]. Oliveira et al. [18] reported a BFS of 20–23 mm for the front ceramic MASs with epoxy composites reinforced with 15, 30, 40 and 50 vol% of fique fabric as the second layer. The SEM analysis showed evidence of fabric yarn pullout and fique fiber stretching, as well as fiber ruptures and matrix cracks.

Regarding the ballistic tests, in addition to BFS determination in the MAS target, two important ballistic parameters can be obtained in stand-alone targets. These are the absorbed impact energy and the limit velocity. Different than the MAS target, in stand-alone targets the target is only the composite plate without the front ceramic [16]. Owing to the high impact velocity of the 7.62 mm projectile, the stand-alone plate is perforated. The residual velocity of the projectile coming out of the plate allows the impact energy absorbed by the composite to be measured. This does not only provide the amount of energy that can be dissipated by the composite alone, but also provides the highest projectile velocity in which the target plate is not perforated; this is known as the limit velocity. In terms of ballistic protection, the limit velocity might indicate the level of ammunition against which the plate could still be used alone as an effective armor. 

This brief report complements previous BFS results from the ballistic tests of fique fabric reinforced epoxy composites as an MAS second layer [18]. For the first time, the absorbed energy and limit velocity in stand-alone targets against the threat of 7.62 mm ammunition have been measured.

## 2. Materials and Methods

### 2.1. Materials

**Figure 1:**
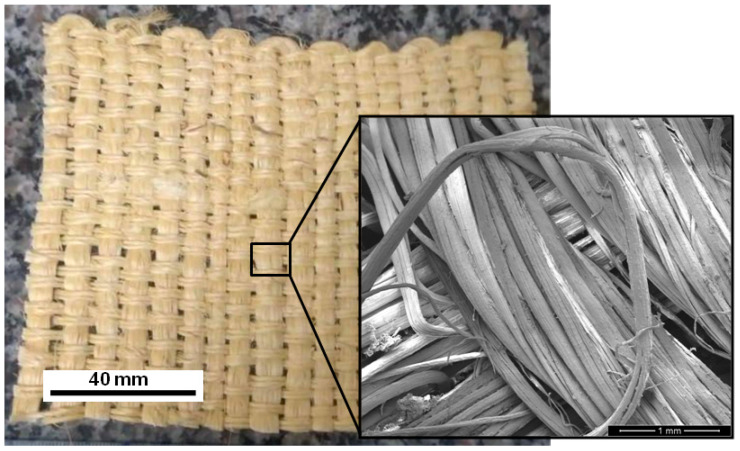
Plain woven fique fabric and microscopic inset of weaved fibers (adapted from [18]).

The fique fabric, illustrated in Figure 1, is plain woven with an areal density of 859 g/cm^2^ in accordance with the NBR 10591/2008 standards [21]. Fique fibers were extracted from the mountain plant *Furcraea andina*, which is native of the Andean regions of Colombia, Ecuador and Peru. In Colombia, fique fibers are commonly available in applications such as textiles for clothes, and fabrics for agricultural products, including sackcloth. The possibility of using fique fabric as reinforcement for polymer composites was reported by Monteiro et al. [22], which motivated its applications as ballistic armor [17,18]. As seen in all previous works, including the present brief report, the fique fabric (Figure 1) was brought from Medellin, Colombia, by one of the authors (H.A.C.L.).

As composite matrix, the commercial epoxy resin diglycidyl ether of the bisphenol-A (DGEBA) was used in a stoichiometric mixture which was hardened with a triethylenetetramine (TETA) catalyst of 13 parts per 100 of resin. Both DGEBA (~100% purity) and TETA (≥96% purity) were produced by Dow Chemical and supplied by Epoxyfiber, Rio de Janeiro, Brazil.

### 2.2. Composite Fabrication

The as-received fique fabric (Figure 1) was cut into 120 × 150 mm pieces that were cleaned in running water to remove impurities, and then dried in a stove at 60 °C for 24 h. The average density of the dried fabric was then measured by a gas pycnometer, Ultrapycnometer 1000 (Quantachrome Instruments, Boynton Beach, FL, USA), and found to be 1.53 g/cm^3^, whereas that of the DGEBA/TETA epoxy was reported as 1.11 g/cm^3^ [22].

Composite plates with 15, 30, 40 and 50 vol% of fique fabric were produced in a steel mold with an internal volume of 180 cm^3^ (150 × 120 × 10 mm^3^) by hand lay-up process. Still fluid DGEBA/TETA epoxy was poured in the mold, filling up the empty space with the precise amount corresponding to the desired volume fractions which were calculated by the fabric and resin densities. Then, the mold was closed, and the composite plate was cured under a 5-tonne load applied by a SKAY hydraulic press, São Paulo, Brazil. Table 1 presents the nomenclature used for the investigated composites.

### 2.3. Stand-Alone Ballistic Tests

Stand-alone ballistic tests were conducted at the Army Assessment Center (CAEx) in Rio de Janeiro, Brazil, using level III 7.62 × 51 mm caliber ammunition with 9.7 g of mass [16].

**Figure 2:**
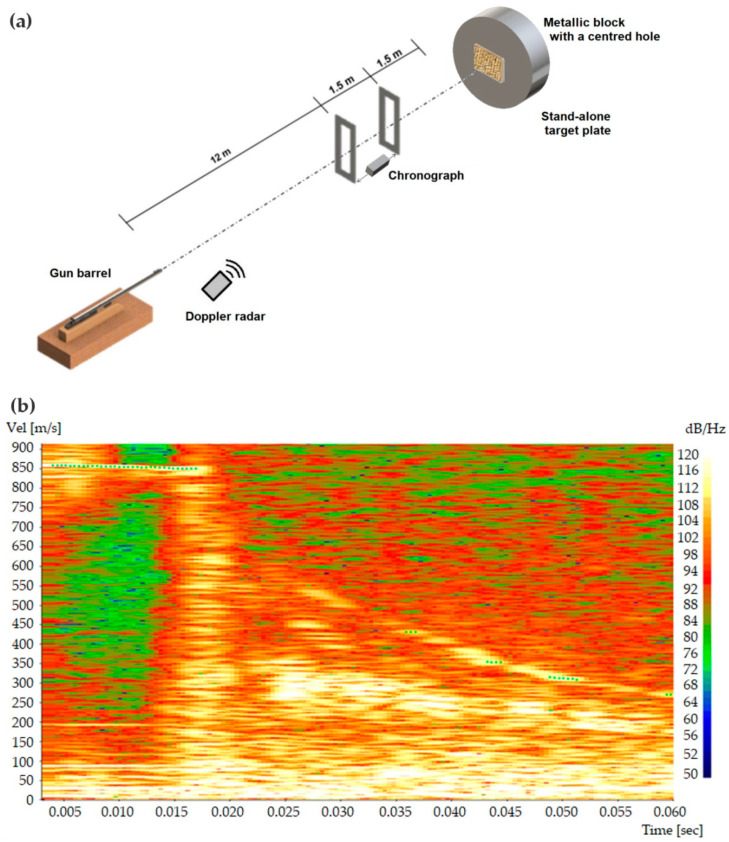
(**a**) Schematic illustration of the CAEx shooting line for stand-alone ballistic test with 7.62 mm ammunition; (**b**) Actual computer-recorded image (radar spectrum) of projectile velocity variation with time also presented.

Figure 2 schematically illustrates the CAEx shooting line. The 7.62 mm projectile was shot from a gun barrel with an initial velocity of 838 ± 15 m/s and hit the stand-alone composite plate, positioned 15 mm from the gun, with a perpendicular (90° angle) trajectory. The optical barrier with the HPI B471 chronograph and model Weibel SL-520P Doppler radar measured both the impact velocity ($v_{i}$) against the target plate and the residual velocity ($v_{r}$) of the projectile leaving the plate after perforation. An actual image (radar spectrum) recording for the projectile velocity (shown in Figure 2b) indicated that the perforation time occurred at around 20 ms for the ballistic tests.

### 2.4. Ballistic Parameters

**Figure 3:**
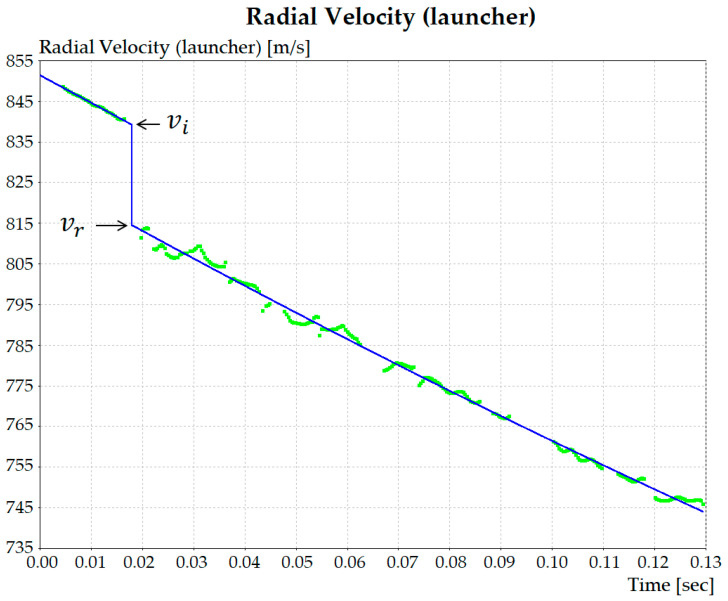
Experimental points obtained from the radar spectrum and the FFT curve fitting.

The absorbed ballistic impact energy ($E_{abs}$) of the stand-alone target plate schematically illustrated in Figure 2 is given by [9]:

(1) $$E_{abs}=\frac{m_{p}\cdot \left(v_{i}{}^{2}-v_{r}{}^{2}\right)}{2}$$

where *m_p_* is the mass, $v_{i}$ and $v_{r}$ are the impact and residual velocities, respectively. The values of $v_{i}$ and $v_{r}$ were calculated for each shot using the program WinDopp^®^, Weibel. The data acquisition by the radar generates a frequency spectrum in the obtained time (Figure 2b), which correlates intensity with velocity by Fast Fourier Transform (FFT) to obtain the velocity curve fitting shown in Figure 3. In this figure, it should be noticed that at ~840 m/s there is an abrupt decrease, which indicates the velocity ($v_{i}$) at the instant of impact. The velocity decrease to ~815 m/s in Figure 3 illustrates the way the residual ($v_{r}$) is measured.

The other important parameter obtained from the stand-alone test is the limit velocity ($V_{L}$) associated with the highest projectile velocity, which still did not perforate the target plate.

According to Morye et al. [23], the $V_{L}$ is a reference velocity that can be related to the maximum level of ammunition [16], which might verify the polymer composite target plate as an effective armor.

(2) $$V_{L}=\sqrt{\frac{2\cdot E_{abs}}{m_{p}}}$$

### 2.5. Weibull Statistical Analysis

The absorbed energy values of seven samples for each different composite investigated in Table 1 were statistically treated using the Weibull method in terms of the cumulative distribution function. A logarithm-based linear expression [18] allows the graphical interpretation of the Weibull parameters:

(3) $$\ln\left[\ln\left(\frac{1}{1-F\left(x\right)}\right)\right]=\beta \cdot \ln\left(x\right)-\left[\beta \cdot \ln\left(\theta \right)\right]$$

where *x* is the absorbed energy, *β* is the Weibull modulus and *θ* is the characteristic energy.

### 2.6. Analysis of Variance (ANOVA)

In order to statistically compare average/standard deviation values of $E_{abs}$ and $V_{L}$, ANOVA was applied to results obtained from the stand-alone ballistic test. The 5% significance level was adopted to verify whether there was a significant difference between the data. A calculated F_calc_ was compared with the tabulated critical F_crit_. In cases where F_calc_ > F_crit_, i.e., *p*-value less than 0.05 (5%), one could conclude with 95% confidence that there is a difference between the experimentally obtained average values. Otherwise, no difference exists if F_calc_ < F_crit_.

### 2.7. Scanning Electron Microscopy (SEM)

SEM images of the fique fabric reinforced epoxy composites after the stand-alone ballistic tests were obtained in a model Quanta FEG 250 FEI microscope operating with secondary electrons at 15 kV. Ballistically fractured composite samples were gold-sputtered for electrical conductivity before SEM analysis.

## 3. Results and Discussion

### 3.1. Stand-Alone Ballistic Tests 

**Figure 4:**
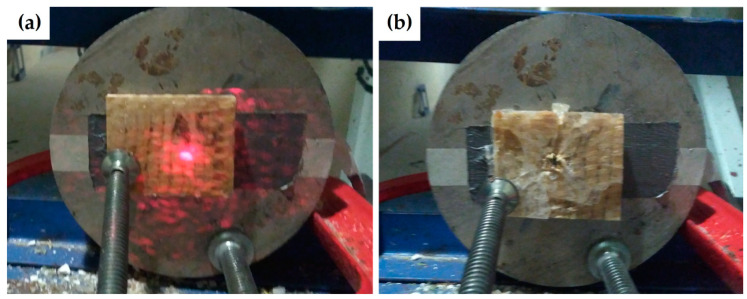
Typical stand-alone ballistic test: (**a**) before shooting, laser beam indicates the direction of 7.62 mm projectile with target plate clamped to a round metallic block; and (**b**) after shooting, there is perforated hole at the center of the plate.

Figure 4 shows the result of a typical stand-alone ballistic test using a 7.62 mm projectile against the fique fabric reinforced epoxy composites. A round metallic block, with a central circular hole with a diameter of 50 mm, was clamped to the back of the target plate to direction the projectile by means of a laser beam (shown in Figure 4a). After the ballistic test, the projectile perforation at the center of the plate (Figure 4b) was revealed as a small dark orifice.

Table 2 presents the main results from the stand-alone ballistic tests, including the determined impact, $v_{i}$, and residual, $v_{r}$, velocities, as well as the absorbed energy, $E_{abs}$, calculated from Equation (1) [9], and the limit velocity, $V_{L}$, from Equation (2) [23]. In this table, not only are the results for the fique fabric reinforced epoxy composites reported, but previous results of the plain epoxy and the Kevlar^TM^ stand-alone plates with the same 10 mm thickness [11,24] are also shown. 

**Figure 5:**
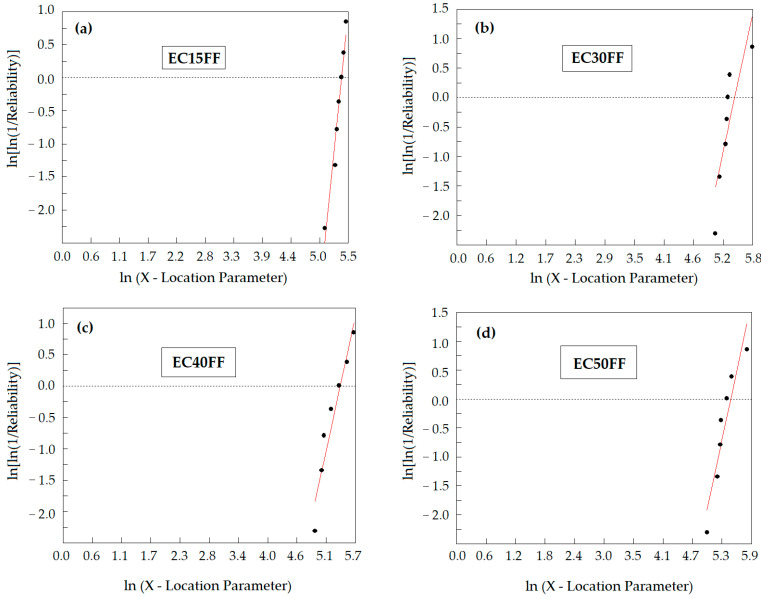
Weibull graphs of the stand-alone absorbed ballistic energy ($E_{abs}\equiv x$) for the investigated epoxy composites with: (**a**) 15 vol%; (**b**) 30 vol%; (**c**) 40 vol%; and (**d**) 50 vol% fique fabric.

Figure 5 shows the Weibull graphs of absorbed energy for the investigated epoxy composites. In these graphs, reasonable linear plots indicate the similar energy absorption mechanisms for each composite. The corresponding mechanisms will be further discussed.

The Weibull parameters calculated from the linear plots in Figure 5 are presented in Table 3.

In this table the relatively good precision of data is indicated by the values of R^2^. However, the relatively low values of the Weibull modulus *β* casts doubts on the possible similar energy absorption mechanisms for all the stand-alone tests of the plates with the same volume fraction of fique fabric. This will be further discussed.

**Figure 6:**
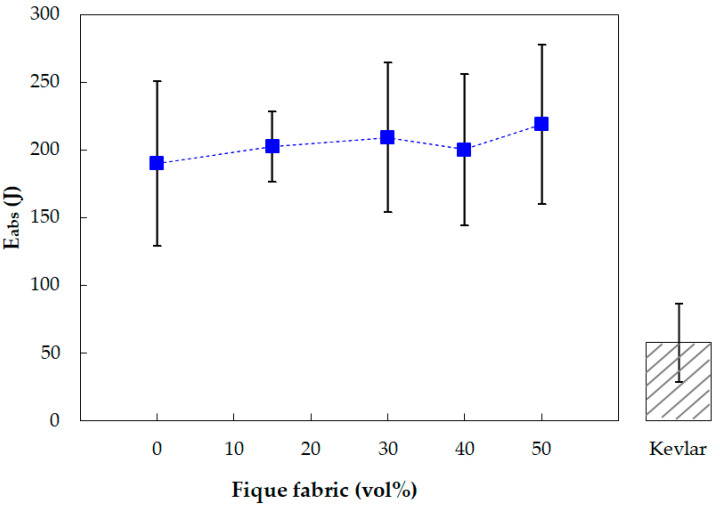
Variation of $E_{abs}$ from the stand-alone test with the volume fraction (vol%) of fique fabric. Reported $E_{abs}$ value for Kevlar^TM^ indicated in adjacent bar [24].

Figure 6 shows the graphic variation of $E_{abs}$ values presented in Table 2 as a function of the fique fabric volume fraction in the epoxy composites. This figure also discloses the value reported for the plain DGEBA/TETA stand-alone epoxy plate with the same 10 mm thickness [11]. The $E_{abs}$ vs. vol% graph in Figure 6 and bar corresponding to the $E_{abs}$ value reported [24] for the Kevlar^TM^ stand-alone plate with the same 10 mm thickness is also shown for comparison.

Within the standard deviations in Figure 6, it is clearly seen that all composites, including the plain epoxy target, have the same $E_{abs}$ values, whereas Kevlar^TM^ has a much lower value.

### 3.2. Analysis of Variance (ANOVA) for Absorbed Energy

Table 4 presents the ANOVA for the $E_{abs}$ obtained in the stand-alone ballistic tests of the fique fabric reinforced epoxy composites shown in Table 2 and Figure 6. In this table, it should be noted that F_calc_ < F_crit_, which guarantees a 95% level of confidence that not only the values (200–219 J) for the investigated composites, but also that (190 J) of the previously reported $E_{abs}$ of the plain epoxy [11], are statistically similar. The reported $E_{abs}$ values within their standard deviation are significantly higher than those reported (58 J) for Kevlar^TM^ [24], shown in the adjacent bar in Figure 6. One may then infer that the plain epoxy and the fique fabric composites more efficiently dissipate the 7.62 mm projectile impact energy as a stand-alone target plate, rather than Kevlar^TM^ with the same thickness. The reason for this favorable ballistic behavior will be further discussed in association with the SEM fracture analysis.

### 3.3. Limit Velocity Discussion 

In principle, the discussion on the calculated results for the limit velocity ($V_{L}$) follows the same methodological reasoning presented in Section 3.1 for the absorbed energy ($E_{abs}$). In fact, it can be seen in Equation (2) that $V_{L}$ is found by a simple square root mathematical transformation of $E_{abs}$. However, a comparison might still be interesting to perform between the $V_{L}$ values in Table 2 and those calculated from the Weibull characteristic energy (*θ*) in Table 3.

**Figure 7:**
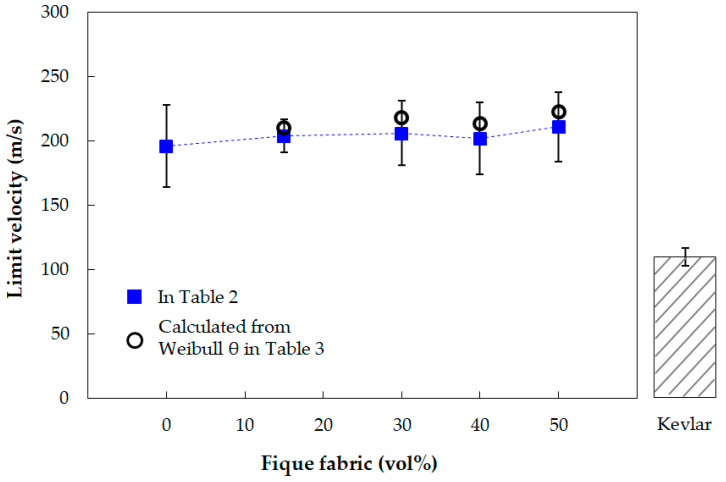
Variation of $V_{L}$ with the volume fraction of fique fabric from values in Table 2 and calculated from Weibull *θ* in Table 3. Reported $V_{L}$ value for Kevlar^TM^ indicated in adjacent bar [24].

Figure 7 shows the variation of $V_{L}$ values presented in Table 2 as a function of fique fabric, including the value previously reported [11] for the plain epoxy. In this figure, the values of $V_{L}$ are also located with open circles which have been calculated with Equation (2) from the Weibull characteristic absorbed energy presented in Table 3.

The graphical results shown in Figure 7 clearly demonstrate that $V_{L}$ remains constant, within 196–223 m/s, with an addition of up to 50 vol% of fique fabric in DGEBA/TETA epoxy matrix. By contrast, the limit velocity of a stand-alone Kevlar^TM^ target plate with the same 10 mm thickness is significantly lower, 109 ± 7 m/s [24]. As mentioned, $V_{L}$ is associated with the highest projectile velocity for which the target plate with a given thickness is not perforated. As such, $V_{L}$ obtained for a given level of ammunition (in the present case a 7.62 mm rifle bullet) serves as an armor design reference. Table 5 compares the limit velocity obtained for the present investigated composites with those reported for epoxy composites incorporated with piassava [25] and mallow [26] natural fibers. 

All results in Table 5, obtained in similar ballistic tests using 7.62 mm ammunition, display comparable values.

In principle, the results of Figure 7 indicate that against the threat of 7.62 mm ammunition, an armor of epoxy reinforced with up to 50 vol% fique fabric could be designed with half the thickness of a Kevlar^TM^ plate. This represents cost and weight reductions as practical advantages. In terms of cost, Oliveira et al. [18] reported that a 150 × 150 mm^2^ ballistic plate with a 10 mm of thickness made of epoxy composite, incorporated with 40 vol% of fique fabric, would cost USD 3.67, whereas the same plate made of Kevlar^TM^ would cost USD 20.61. These findings present the same plate made of Kevlar^TM^ as more than five times the price of the fique fabric composite. In addition, there are environmental and societal benefits associated with the use of natural fiber and fabrics [27]. However, integrity is another point to be considered in armor design [16].

It was previously found [11] that a plain epoxy is associated with a relatively high limit velocity (196 m/s in Table 2) but was found to completely shatter after the impact of a 7.62 mm projectile. In comparison, a stand-alone Kevlar^TM^ plate kept its integrity [24]. Regarding the investigated fique fabric composites, the question remains as to whether they withhold the necessary integrity to withstand subsequent shooting as required by the standards.

### 3.4. Composites Integrity and Failure Analyses

**Figure 8:**
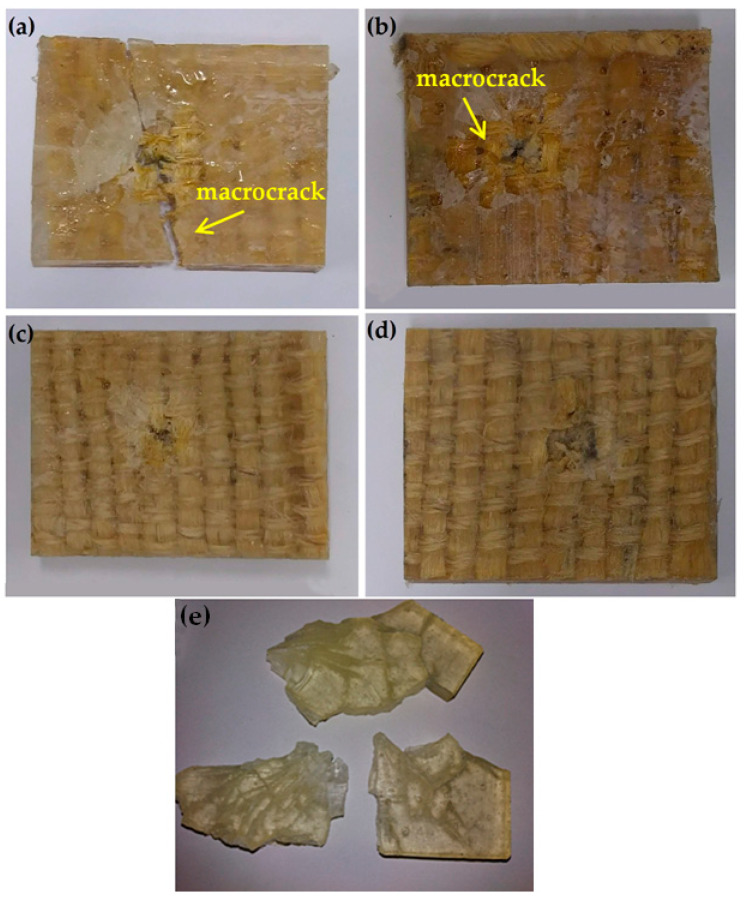
Stand-alone epoxy composite plates after the ballistic test against 7.62 mm ammunition: (**a**) 15 vol%; (**b**) 30 vol%; (**c**) 40 vol%; (**d**) 50 vol% fique fabric; and (**e**) plain epoxy plate.

Figure 8 illustrates the typical fique fabric epoxy composite stand-alone plates after the ballistic test. Composites with 15 vol% (Figure 8a) and 30 vol% (Figure 8b) of fique fabric display visible damages in the form of macrocracks. Similar to the shattered plain epoxy, these composites with 15 and 30 vol% of fique fabric are ruled out as possible armor, even with the thickness large enough to be associated with a limit velocity of ~840 m/s, corresponding to a 7.62 mm projectile. On the other hand, composites with 40 vol% (Figure 8c) and 50 vol% (Figure 8d) did not develop macrocracks, and might withstand subsequent shootings without open spaces that allow the easy passage of the projectile. All of the composites in Figure 8 show a central hole from where the projectile perforated the target plate. Figure 8 also presents the plain epoxy plate completely fractured after the ballistic impact in the stand-alone test (Figure 8e).

**Figure 9:**
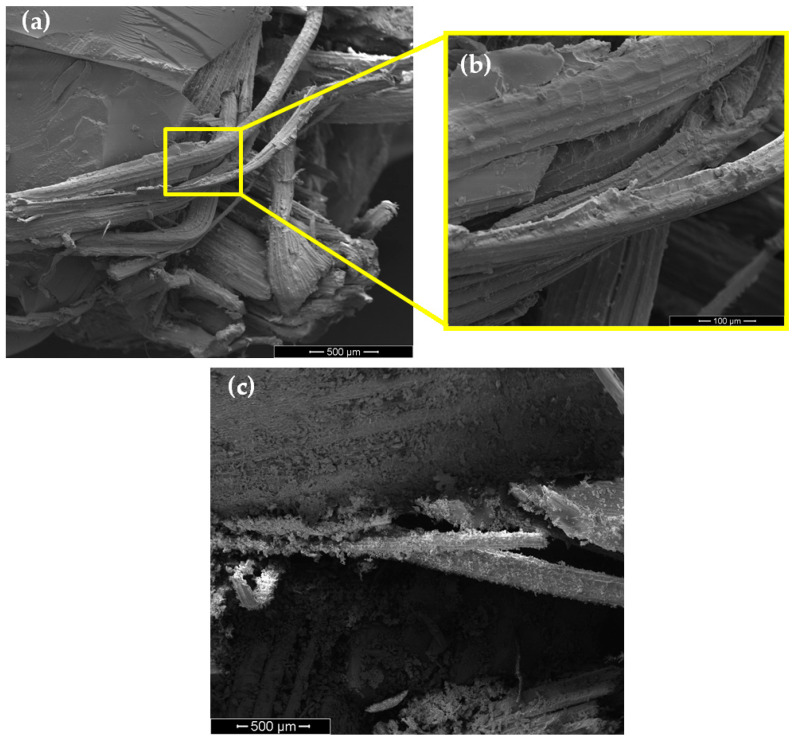
SEM image of the damage in the epoxy composite with 15 vol% of fique fabric (**a**,**b**); and with 40 vol% of fique fabric (**c**).

As shown in Figure 8, both of the 15 and 30 vol% fabric epoxy composites display damage by macrocracks that hinder their application as ballistic armor. The SEM observation with the high magnification of a macrocrack in the epoxy composite with 15 vol% of fique fabric is shown in Figure 9a. In this figure, a clear separation exists between fibers in the fique fabric and the epoxy matrix. This separation on a large scale constitutes evidence of delamination, which could be associated with the main damage caused to the composite structure by the 7.62 mm projectile impact. Another consequence of this impact is the fique fiber rupture causing microfibrillation, illustrated in the inset of Figure 9b. On the other hand, the SEM analysis of the damaged regions close to the bullet penetration hole in the 40 vol% fique fabric composite in Figure 9c showed only microcracks and broken fibers. No evidence of lamination could be found in this composite with a higher volume fraction of fique fabric. These different mechanisms occurring in the fique fabric composites might justify the relatively low values of the Weibull moduli in Table 3.

With the exception of the central perforated hole (Figure 8c,d), no apparent visible damage occurred for the 40 and 50 vol% fique fabric composites. One may infer that volume fractions above 30 vol% fique fabric composites prevent delamination and maintain the composite integrity, which is required for protection against subsequent shooting [16]. Therefore, this brief report complements the previous work [18] in which the same fique fabric reinforced epoxy composites were found to provide effective protection as an MAS second layer against 7.62 mm ammunition. Herein, however, it is revealed that, as stand-alone armor, only epoxy composites with an amount of fique fabric above 30 vol% will guarantee effective ballistic protection. These composites were also found to have superior ballistic performance in terms of absorbing impact energy and limiting velocity, as compared to a Kevlar^TM^ armor plate with the same thickness.

## 4. Summary and Conclusions

The plain woven fabric made of fique fibers, extracted from the leaves of *Furcraea andina*, was used in amounts of 15, 30, 40 and 50 vol% of epoxy matrix composite reinforcement and ballistic-tested as a stand-alone target plate with 10 mm thickness against 7.62 mm rifle ammunition.

The absorbed impact energy ($E_{abs}$) of 200–219 J, including the already reported value of 190 J for the plain epoxy, are found to be equal by the ANOVA test with a 95% level of confidence. These values are significantly higher than the $E_{abs}$ of 58 J reported for the aramid fabric (Kevlar^TM^) target plate with the same thickness.

The limit velocity ($V_{L}$) calculated from both the absorbed impact energy (202–211 m/s) and the Weibull characteristic parameter (210–223 m/s), including the previously reported value of 196 m/s for the plain epoxy, are practically similar within the standard deviations. On the contrary, the $V_{L}$ reported for the Kevlar^TM^ 10 mm thick stand-alone target plate of 109 m/s is marked lower.

The visible macrocracks developed in the 15 and 30 vol% fique fabric composites compromise their application as 10 mm thick armor plates. In the case of the plain epoxy, the plate was reported to be completely shattered. As for the 40 and 50 vol%, only the projectile perforation hole was visible, which indicated that the integrity was maintained.

The SEM analysis revealed that the main damage responsible for the macrocracks is associated with fiber/matrix delamination and fiber rupture causing microfibrillation.

As stand-alone armor, only epoxy composites reinforced with more than 30 vol% of fique fabric guarantee superior ballistic protection compared to Kevlar^TM^.

The present ballistic results disclose an optimistic prospective for future research on natural fabric polymer composites as a possible substitute for more expensive and currently used synthetic laminates in personal armor protection.

## Figures and Tables

**Table 1:** Nomenclature adopted for the composites.

Nomenclature	Material
EC15BF	Epoxy composite with 15 vol% fique fabric
EC30FF	Epoxy composite with 30 vol% fique fabric
EC40BF	Epoxy composite with 40 vol% fique fabric
EC50BF	Epoxy composite with 50 vol% fique fabric

**Table 2:** Results of computer-recorded velocities and calculated parameters from 7.62 mm ballistic stand-alone tests for fique fabric reinforced epoxy composites, as well as previously reported results for plain epoxy and Kevlar^TM^.

Stand-Alone 10 mm Thick Plate Target	$v_{i}$ (m/s)	$v_{r}$ (m/s)	$E_{abs}$ (J)	$V_{L}$ (m/s)	Ref.
EC15FF	839 ± 7	814 ± 6	203 ± 26	204 ± 13	PW
EC30FF	840 ± 8	813 ± 8	209 ± 55	206 ± 25	PW
EC40FF	837 ± 4	812 ± 10	200 ± 56	202 ± 28	PW
EC50FF	843 ± 4	816 ± 5	219 ± 59	211 ± 27	PW
DGEBA/TETA epoxy	850 ± 2	827 ± 6	190 ± 61	196 ± 32	[11]
Kevlar (ply of aramid fabric)	848 ± 6	841 ± 7	58 ± 29	109 ± 7	[24]

PW: Present work.

**Table 3:** Weibull parameters related to stand-alone absorbed ballistic energy of fique fabric reinforced epoxy composites associated with graphs in Figure 5.

Stand-Alone Composite Plate Target	*β*	*θ* (J)	R^2^
EC15FF	7.85	214.6	0.96
EC30FF	4.01	231.8	0.73
EC40FF	3.78	221.7	0.93
EC50FF	4.05	241.2	0.88

**Table 4:** ANOVA of $E_{abs}$ in the stand-alone ballistic test of fique fabric reinforced epoxy composites and plain epoxy.

Variation Causes	Sum of Squares	DF	Mean of Squares	F_calc_	F_crit_	*p*-Value
Treatment	2304.48	4	576.12	0.22	2.74	0.93
Residual	69,215.74	26	2662.14			
Total	71,520.22	30				

**Table 5:** Limit velocity for the fique fabric epoxy composites compared to piassava and mallow fiber epoxy composites.

Epoxy Composite	$V_{L}$ (m/s)	Reference
15 vol% fique fabric	204 ± 13	PW
30 vol% fique fabric	206 ± 25	PW
40 vol% fique fabric	202 ± 28	PW
50 vol% fique fabric	211 ± 27	PW
10 vol% piassava fiber	236 ± 8	[25]
20 vol% piassava fiber	200 ± 9	[25]
30 vol% piassava fiber	202 ± 7	[25]
40 vol% piassava fiber	198 ± 6	[25]
50 vol% piassava fiber	204 ± 2	[25]
30 vol% mallow fiber	231 ± 18	[26]

PW: Present work.
